# Hepatic alveolar echinococcosis infection induces a decrease in NK cell function through high expression of NKG2A in patients

**DOI:** 10.3389/fimmu.2025.1563248

**Published:** 2025-05-12

**Authors:** Abuduaini Abulizi, Talaiti Tuergan, Paizula Shalayiadang, Chuanshan Zhang, Ruiqing Zhang, Tiemin Jiang, Qiang Guo, Hui Wang, Liang Li, Renyong Lin, Yingmei Shao, Tuerganaili Aji

**Affiliations:** ^1^ Hepatobiliary & Hydatid Disease Department, Digestive & Vascular Surgery Center, First Affiliated Hospital of Xinjiang Medical University, State Key Laboratory of Pathogenesis, Prevention and Treatment of High Incidence Diseases in Central Asia, Urumqi, China; ^2^ World Health Organization (WHO) Collaborating Center on Prevention and Management of Echinococcosis, Clinical Medicine Institute, First Affiliated Hospital of Xinjiang Medical University, Urumqi, China; ^3^ State Key Laboratory on Pathogenesis Prevention & Treatment of High Incidence Diseases in Central Asia, Xinjiang Medical University, Urumqi, China

**Keywords:** hepatic alveolar echinococcosis, NK cell, NKG2A, immune exhaustion, IFN-γ

## Abstract

*Echinococcus multilocularis* larval tapeworm infection in humans is considered a serious public health issue. The immune interaction between parasites and their hosts is extremely important. NK cells are known innate immune cells that play important roles during infection and tumour progression. However, the possible role of NK cells in hepatic alveolar echinococcosis is not completely clear. In this study, we investigated the functional decrease in NK cells in hepatic alveolar echinococcosis (AE) patients.

**Methods:**

Using human liver tissue samples from 10 patients with hepatic AE, flow cytometry was used to detect the expression of NKG2A molecules on the surface of NK cells, and the correlations between NKG2A+ expression and lesion size, alkaline phosphatase (ALP) levels in close lesion tissues (CLTs) and distal lesion tissues (DLTs) in the liver, and the secretion of functional molecules by NKG2A+ NK cells were analysed.

**Results:**

The expression of NKG2A on CD56dim and CD56bright NK cells in DLTs and CLTs revealed that the percentage of NKG2A+CD56dim NK cells in CLTs was significantly greater than that in DLTs. There was a negative correlation between the expression of NKG2A on NK cells in the CLT and alkaline phosphatase. Additionally, we analysed IFN-γ, TNF-α, granzyme B, and perforin production in NK cells. There was a significant reduction in IFN-γ production in CLTs compared with DLTs. There is a negative correlation between IFN-γ production levels and NKG2A expression in NK cells from the CLT. The capacity of NKG2A+ NK cells from CLT regions to produce IFN-γ and granzyme B was also significantly decreased. In contrast, the perforin level produced by NKG2A+ NK cells was much greater than that produced by NKG2A− NK cells. We also analysed the correlation between the ratio of the NKG2A expression area in CLT and DLT tissues and the PET–CT value and found a positive correlation between NKG2A expression and the PET–CT value.

**Conclusion:**

The increased expression of NKG2A in NK cells induced a reduction in IFN-γ production, and the increased expression of NKG2A may improve lesion activity and fibrosis, which may be helpful for treating hepatic alveolar echinococcosis via immunity.

## Introduction

Human alveolar echinococcosis (AE) is a potentially lethal zoonosis caused by the cestode *Echinococcus multilocularis* (1); it is prevalent mainly in Western China, the Middle East, and Central Europe (2, 3). China accounts for 91% of the global AE burden every year; thus, sustained efforts have been made to prevent, control, and manage this disease (2). Humans can contract AE through contaminated food or water, and the liver is the primary infected organ. If not treated in time, the disease infiltrates and consequently leads to the critical involvement of vasculature and to jaundice, cirrhosis, and other clinical symptoms, which can result in liver failure or even death (4). The fatality rate of untreated or inadequately treated patients with human alveolar echinococcosis is 90% at 10–15 years after diagnosis (4, 5). Hence, it is regarded as a “parasitic cancer” (6).

The immune mechanism of *E. multilocularis* larvae is important for self-healing or persistent chronic infection after they enter the liver. In hepatic AE patients and animal models, long-term parasitism by larvae is mediated mainly by regulatory T cells and related cytokines, such as IL-10 and TGF-β (7). Our recent studies demonstrated the potential importance of the remaining Th subsets, such as Th17, Treg, and Th9, in *E. multilocularis* infection (8). Our data indicated that *E. multilocularis* can induce T-cell exhaustion through the inhibitory receptor TIGIT and that blocking this checkpoint may reverse the functional impairment of T cells and represent a possible approach to immunotherapy against AE (9). In addition, *E. multilocularis* vesicular fluid increases PD-L1 and CTLA-4 expression in T cells (10).

As important innate immune cells, NK cells constitute the first line of defence against infection and tumours. Many related studies have shown its special role in the tumour progression of liver cancer patients (11). The ability of intrahepatic NK cells to secrete IFN-γ and induce cytotoxicity is decreased significantly in hepatocellular carcinoma patients (12). The intracellular parasite *Toxoplasma gondii* impairs the ability of NK cells to recognize target cells and reduces the secretion of IFN-γ in the host (13). Malaria may increase the ability of NK cells to secrete IFN-γ via KIR/HLA molecules in early-stage infection (14), and *Leishmania* can inhibit the proliferation of NK cells (15). Extracellular parasites: When the percentage of NK cells decreases, the expression of NKG2D, CD69, and Ly49A is upregulated in individuals infected with *Angiostrongylus cantonensis* (16), and *Schistosoma japonicum* inhibits liver fibrosis by activating liver NK cells and increasing IFN-γ secretion (17). Several related studies have shown that in patients with hepatic alveolar echinococcosis, the activity of NK cells in the peripheral blood is significantly downregulated, and *E. multilocularis* vesicular fluid may inhibit the activation and proliferation of NK cells in mice (18).

NKG2A is specifically expressed on the surface of some lymphocytes, such as NK cells, T cells, and NKT cells, and transduces inhibitory signals (19). High expression of NKG2A induces functional downregulation of NK cells and is related to poor prognosis in patients with hepatic cellular carcinoma (20). Currently, the newest research reports that NKG2A is a new checkpoint, and blocking NKG2A with monalizumab could promote the antitumour immune activity of CD8+ T cells and NK cells, which could be used as a supplement to the first generation of cancer immunotherapy (11). Our recent study demonstrated that a reduction in NK cell frequency and increased NKG2A may result in low cytotoxic activity through decreased IFN-γ secretion during *E. multilocularis* infection (18). Therefore, studies on the correlation between the expression of NKG2A on NK cells and the pathological and clinical parameters of hepatic AE are very important.

However, some research has focused on immune interactions in *E. multilocularis* infection. NK cells are involved in *E. multilocularis* infection both *in vitro* and *in vivo*. However, very little is known regarding the possibility of immune dysfunction in hepatic AE patients. In this study, we demonstrated NKG2A expression in hepatic NK cells and its functional exhaustion, such as decreased IFN-γ production and decreased cellular cytotoxicity. These findings may indicate the existence of a negative regulatory mechanism in exhausted NK cells as a result of the increased expression of the inhibitory receptor NKG2A in hepatic AE patients.

## Patients and methods

Liver tissues from 36 hepatic AE patients and fresh hepatic AE liver tissue samples were obtained from 10 hepatic AE patients who underwent radical hepatectomy between 2012 and 2015 in the Department of Hepatobiliary and Echinococcosis Surgery Department of the First Affiliated Hospital of Xinjiang Medical University (21–23). In accordance with the location of surgical samples from patients with hepatic AE, the liver tissue adjacent to the lesion (0.5 cm) and its volume of approximately 2 cm × 2 cm × 2 cm were selected as the liver close lesion tissue (CLT), and the corresponding normal liver tissue approximately 3–5 cm away from the lesion was taken as the liver distal lesion tissue (DLT). All fresh tissues were used for phenotypic analysis, and most of them were also used for intracellular cytokine analysis if they had a sufficient number of cells. All patients provided written informed consent in accordance with the Declaration of Helsinki. The protocols for all study cohorts were approved by the Ethics Board of Xinjiang Medical University.

### Flow cytometry

Liver tissue-infiltrating leukocytes were obtained as previously described (9). To digest the samples, they were cut into small pieces and digested in RPMI 1640 (HyClone Laboratories, Logan, UT, USA) supplemented with collagenase IV (10 mg/mL, Sigma-Aldrich Corp., St. Louis, MO, USA) and DNase I (33.3 mg/mL, Sigma-Aldrich, USA) at 37°C for 1–2 h. The peripheral lymphocytes, liver tissue-infiltrating leukocytes, and NK cells from the *in vitro* cultures were stained with fluorochrome-conjugated Abs and then analysed through flow cytometry (24). Abs against the following proteins were used for staining: CD3 (UCHT1), CD56 (HCD56), CD16 (3G8), CD69 (FN50), NKG2D (1D11), IFN-γ (4S.B3), TNF-α (MAb11), perforin (dG9), and granzyme B (GB11) (BioLegend, USA) and NKG2A (131411) (R&D Systems, Minneapolis, MN, USA). The stained cells were analysed using an LSRFortessa flow cytometer (Becton Dickinson, Franklin Lakes, NJ, USA), and the data were analysed using the FlowJo analysis software V10 (Treestar, Woodburn, OR, USA).

### Immunohistochemistry

The paraffin-embedded sections were dewaxed in xylene and rehydrated with distilled water. Following incubation with antibodies against human NKG2A (PA5-21949, Thermo Fisher, Waltham, MA, USA), adjacent sections were stained with the DAB Peroxidase Substrate Kit (SK-4100) (Vector Laboratories, Burlingame, CA, USA). Positive and negative controls were tested before formal staining. The Olympus Optical Microscope cellSens scanning software (Olympus, Tokyo, Japan) was used to perform scanning analysis, and the positive staining surface around the lesion was calculated (25).

### NK cell purification

Peripheral blood was obtained from healthy controls, and purified NK cells were enriched from whole blood via RosetteSep™ Human NK Cell Cocktail (50 μL/mL) (STEMCELL Technologies, Vancouver, BC, Canada). The cells were incubated in medium alone or with recombinant human IL-12 p40 (200-12p40) (10 ng/mL; PeproTech, Cranbury, NJ, USA, USA) plus recombinant human IL-15 (200-15) (10 ng/mL; PeproTech, USA), with IL-12+IL-15 plus recombinant human TGF-β1 (1 ng/mL; PeproTech, USA), or with IL-12+IL-15 plus EMP (*E. multilocularis* protein). The protein was extracted and quantified after grinding *E. multilocularis*. NK cells were stimulated for 24 h at 37°C in an atmosphere of 5% CO_2_. NK cell viability was evaluated via the trypan blue exclusion method and analysed via flow cytometry after incubation.

### Statistical analysis

Statistical analysis was performed using GraphPad Prism 7.0 (GraphPad Software, San Diego, CA, USA). The Wilcoxon non-parametric statistical test or Mann–Whitney non-parametric statistical test was used when there were more than two groups. *p* < 0.05 was considered statistically significant. *p*-Values are presented as ^*^ *p* < 0.05, ^**^ *p* < 0.01, ^***^ *p* < 0.001, and ^****^ *p* < 0.0001.

### Increased NKG2A expression in NK cells from the close lesion tissue of hepatic AE patients

**Figure 1:**
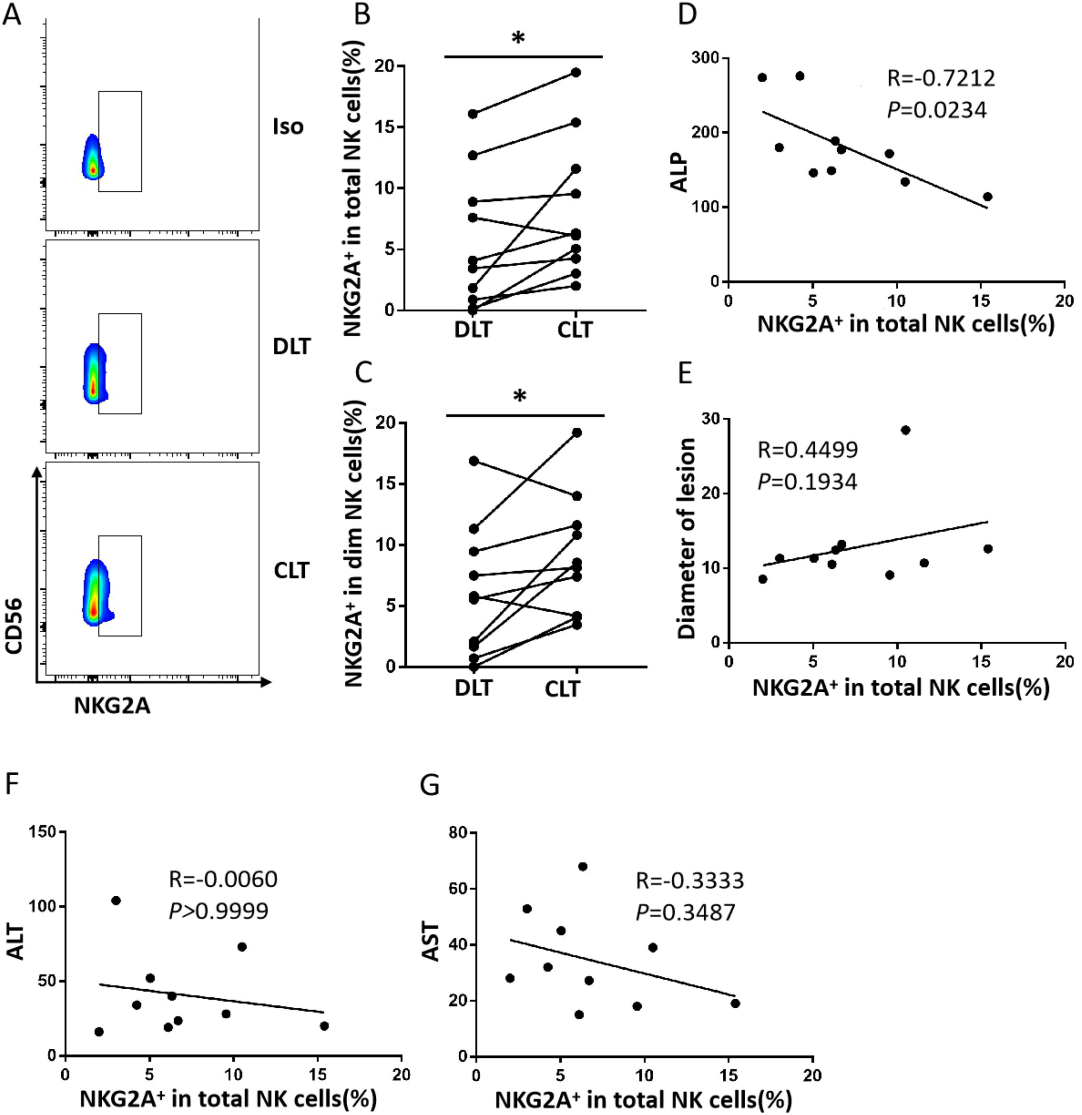
The frequency of close lesion tissue NKG2A+ NK cells is increased in hepatic AE. **(A)** Representative NKG2A expression in different liver NK cell subsets from DLTs and CLTs from hepatic AE patients. **(B)** NKG2A expression in NK cells from paired DLTs and CLTs from hepatic AE patients (N = 10, Wilcoxon non-parametric statistical test). **(C)** Percentages of NKG2A+CD56dim NK cells in paired DLTs and CLTs from patients with hepatic AE (n = 10, Wilcoxon non-parametric statistical test). **(D–G)** Correlations between NKG2A expression in hepatic NK cells from CLTs and the serum Alkaline Phosphatase (ALP), ALT, and AST levels and lesion diameters in hepatic AE patients. Spearman’s correlation coefficients are shown. (P values are presented as *P < 0.05). AE, alveolar echinococcosis; DLTs, distal lesion tissues; CLTs, close lesion tissues; ALT, alanine aminotransferase; AST, aspartate aminotransferase.

To investigate the expression of NKG2A in intrahepatic NK cells, we analysed the CLT and DLT of liver AE lesions. As shown in **Figures 1A–C**, the percentage of NKG2A+ NK cells in the CLT was significantly greater than that in the DLT. As previously demonstrated (26), we analysed the expression of NKG2A on CD56dim and CD56bright NK cells in DLT and CLT and reported that the percentage of NKG2A+CD56dim NK cells in CLTs was significantly greater than that in DLTs. There was a negative correlation between the expression of NKG2A on NK cells in the CLT and alkaline phosphatase (ALP) (*p* = 0.0234, r = −0.7212) (**Figure 1D**), but there was no correlation between alanine aminotransferase (ALT) and aspartate aminotransferase (AST) levels (**Figures 1E–G**). These results suggest that the percentage of NKG2A+ NK cells in the CLT of hepatic AE patients was significantly increased, which was mainly manifested by the upregulation of NKG2A expression on CD56dim NK cells.

### Increased expression of NKG2A in NK cells induced a significant reduction in IFN-γ production in close lesion tissue compared with distal lesion tissue from hepatic AE patients

**Figure 2:**
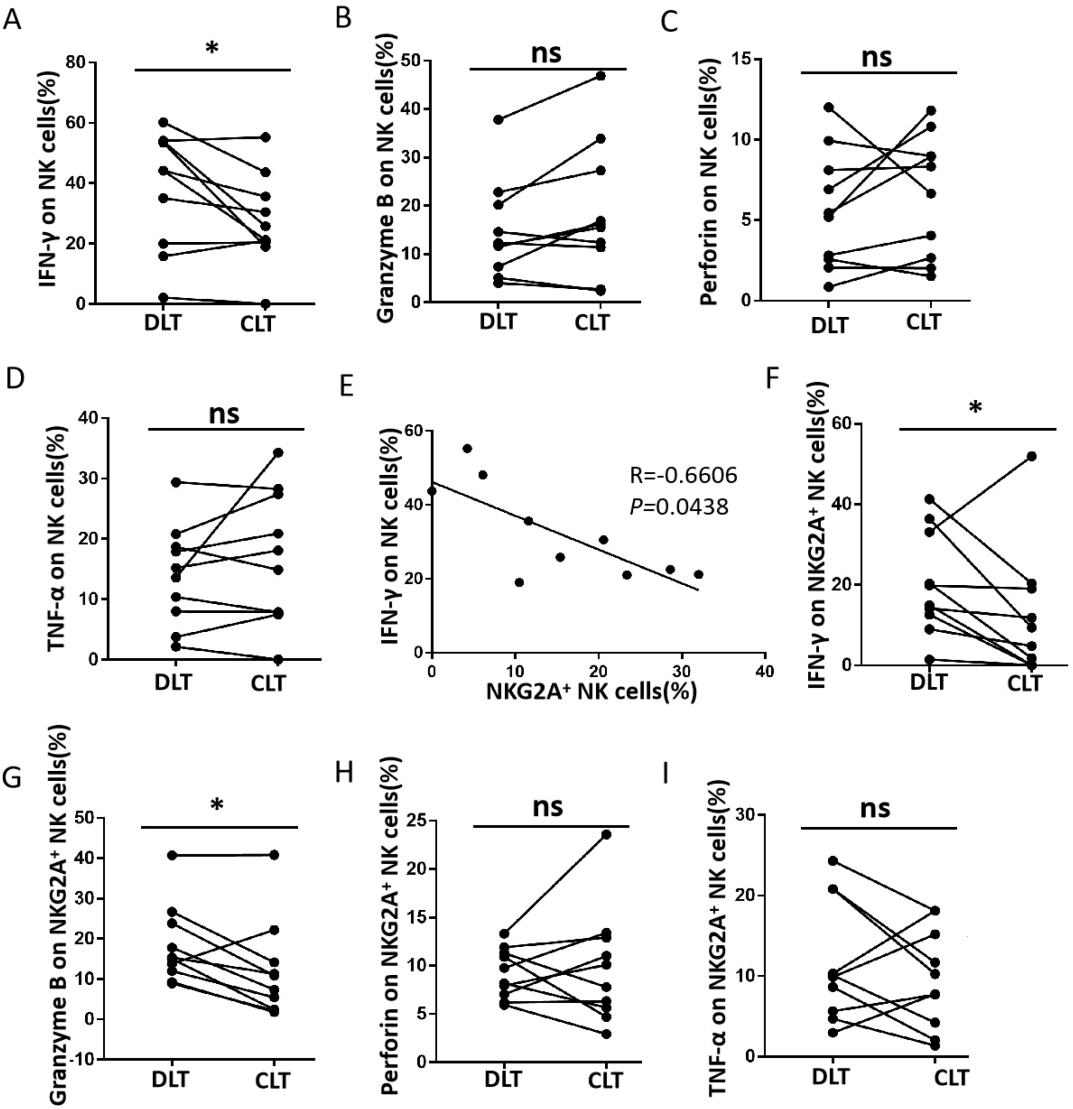
Functional impairment of CLT NK cells from hepatic AE patients. **(A–D)** The levels of IFN-γ, granzyme B, perforin, and TNF-α produced by NK cells in the CLT and DLT regions in hepatic AE patients (n = 10, Wilcoxon non-parametric statistical test). **(E)** Analysis of the correlation between IFN-γ levels and the percentage of NKG2A+ NK cells in the tissue samples. Each dot represents a single region CLT from a hepatic AE patient (Spearman’s correlation test). **(F–I)** The levels of IFN-γ, granzyme B, perforin, and TNF-α produced by NKG2A+ NK cells in CLT regions in hepatic AE patients (n = 10, Wilcoxon non-parametric statistical test). (P values are presented as *P < 0.05), no significant different (ns). CLT, close lesion tissue; AE, alveolar echinococcosis; DLT, distal lesion tissue.

**Figure 3:**
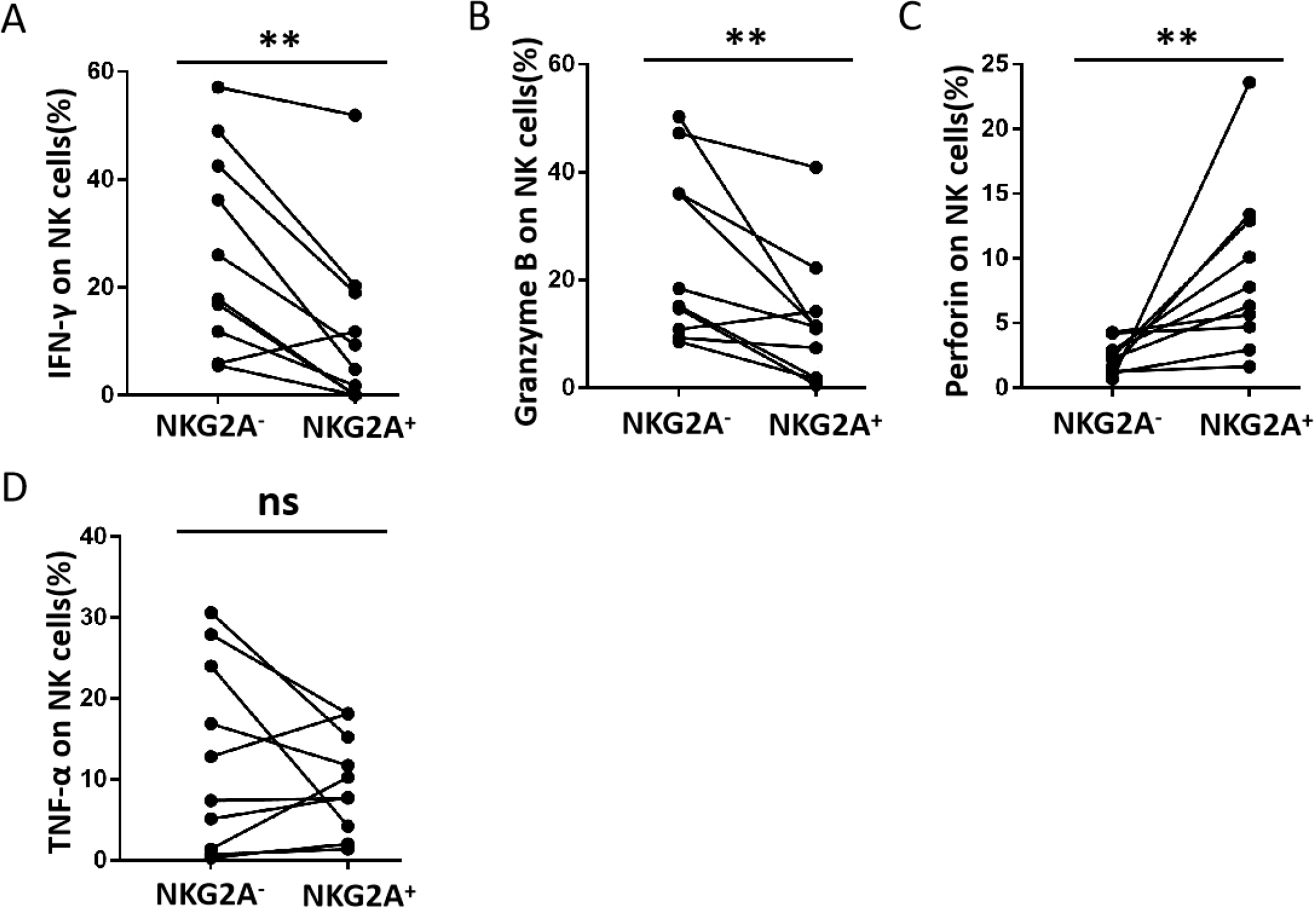
The ability of NKG2A+ and NKG2A− NK cells in CLT from hepatic AE patients to produce cytokines. **(A–D)** The levels of IFN-γ, granzyme B, perforin, and TNF-α produced by NKG2A+ NK and NKG2A− NK cells in CLT regions in hepatic AE patients (n = 10, Wilcoxon non-parametric statistical test). (P values are presented as **P < 0.01), no significant different (ns). CLT, close lesion tissue; AE, alveolar echinococcosis.

The function of NK cells is affected by combining both activating receptors and inhibitory receptors. According to previous studies (27), NKG2A-induced phenotypic changes may be accompanied by functional alterations in NK cells. Therefore, we analysed IFN-γ, TNF-α, granzyme B, and perforin production in NK cells (**Figure 2**). There was a significant reduction in IFN-γ production in the CLT compared with DLT (**Figure 2A**). However, there was no significant difference in granzyme B, perforin, or TNF-α levels (**Figures 2B–D**). A negative correlation was detected between IFN-γ production and NKG2A expression in NK cells from the CLT (**Figure 2E**). In addition, the capacity of NKG2A+ NK cells from CLT regions to produce IFN-γ and granzyme B was also significantly decreased (**Figures 2F, G**). However, the ability of NKG2A+ NK cells to produce TNF-α and perforin was not significantly different between the CLT and the DLT (**Figures 2H, I**). In addition, the ability of NKG2A+ NK cells to secrete IFN-γ and granzyme B was, surprisingly, significantly lower than that of NKG2A− NK cells (**Figures 3A, B**). In contrast, NKG2A+ NK cells produced much higher perforin levels than NKG2A+ NK cells did (**Figure 3C**), but there is there was no significant difference produced TNF-α levels (**Figure 3D**). These findings suggest that the increased expression of NKG2A on NK cells in the CLT decreased the ability of NK cells to secrete IFN-γ.

### Higher NKG2A expression is correlated with lesion activity and fibrosis in hepatic AE patients

**Figure 4:**
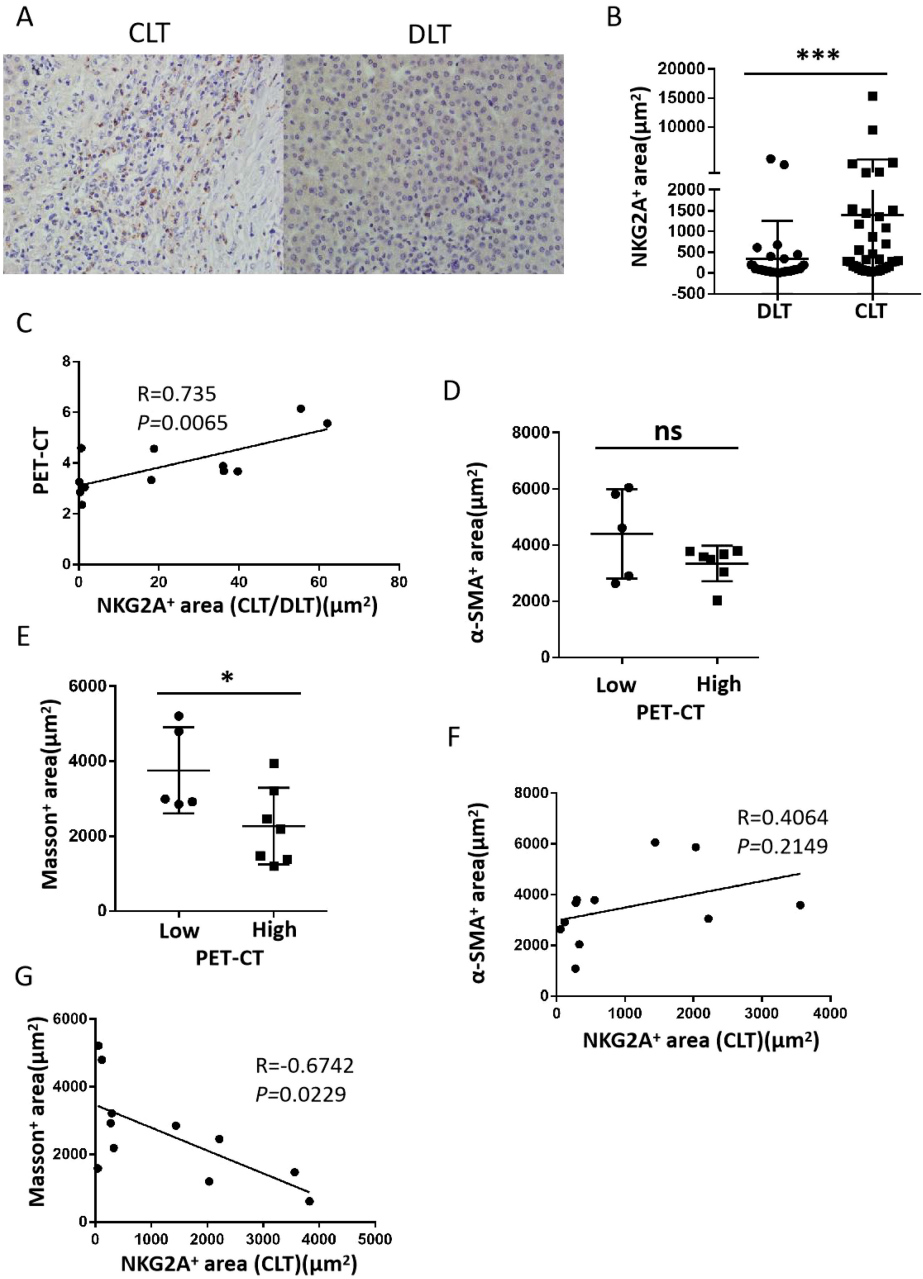
The expression of NKG2A and fibrosis around lesions in CLT regions from hepatic AE patients. **(A)** Representative micrographs showing NKG2A+ cells in the CLT and DLT of hepatic patients. Original magnifications, ×10, ×40; bar D 100 mm. **(B)** Cumulative data are shown (Mann–Whitney unpaired non-parametric statistical tests). **(C)** Correlation between NKG2A+ expression and the PET–CT value (SUVmax) in the CLT region from hepatic AE patients (N = 12) (Spearman’s correlation coefficient (r) and (p) values are shown). **(D, E)** α-SMA immunohistochemical staining revealed positive expression of hepatic stellate cells, and Masson staining revealed the collagen area (fibrotic area) around the lesion in high-activity and low-activity lesions from the hepatic AE lesion. **(F, G)** Correlations between positive expression of hepatic stellate cells with NKG2A+ expression area and fibrosis prelesion area with NKG2A+ expression [Spearman’s correlation coefficients (r) and (p) values are shown]. (P values are presented as *P < 0.05; ***P < 0.001), no significant different (ns). CLT, close lesion tissue; AE, alveolar echinococcosis; DLT, distal lesion tissue.

After the expression of NKG2A in CLT and DLT tissues from 36 patients with hepatic AE was examined, our results suggested that the expression of NKG2A was significantly increased in the CLT (**Figures 4A, B**). We also analysed the correlation between the ratio of the NKG2A expression area in the CLT and DLT tissues (CLT/DLT) and the PET–CT value (SUVmax) and found a positive correlation between NKG2A expression and the PET–CT value (SUVmax) (*p* = 0.0065, R = 0.735) (**Figure 4C**). In this study, according to liver tissue samples from patients with hepatic AE who underwent PET–CT examination, Masson staining was used to detect the collagen area (fibrotic area) around the lesion, and α-SMA immunohistochemical staining was used to detect the positive expression of hepatic stellate cells. A PET–CT (SUVmax) value greater than or equal to 3.0 was included in the high-activity lesion group, and a value less than 3.0 was included in the low-activity lesion group. The results revealed that the area of fibrosis around lesions was significantly lower in high-activity lesions than in low-activity lesions (**Figures 4D, E**), and a negative correlation existed between the NKG2A+ expression area and the fibrosis prelesion area (**Figures 4F, G**) (these results need to be further verified by expanding the sample size). These results suggest that the expression of NKG2A in the CLT was upregulated and was positively correlated with the activity of the lesion. Moreover, the greater activity of the lesion and the lower degree of fibrosis around the lesion result in the lesion not being fully limited by the fibrous layer, which induces faster growth of the hepatic AE lesion.

### Compared with those of CD56bright NK cells, the percentages of NK cells are predominantly decreased on CD56dim NK cells

**Figure 5:**
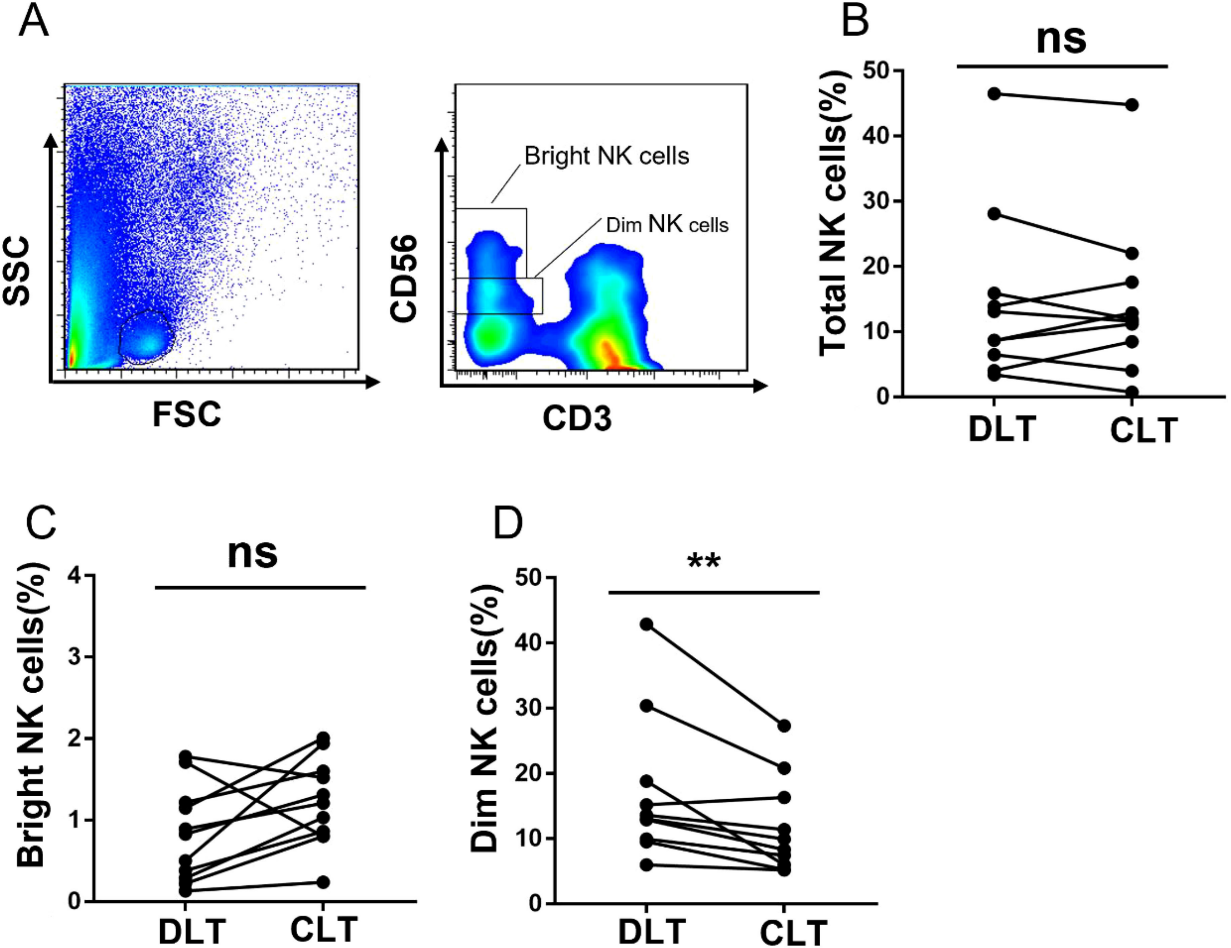
The subset of NK cells in CLT is decreased, and those in hepatic AE patients are CD56dim NK cells. **(A)** The gating strategy for the CLT used to analyse CD56bright NK cells and CD56dim NK cells via flow cytometry. **(B)** The percentages of total NK cells in the CLTs and DLTs from hepatic AE patients (N = 10, Wilcoxon non-parametric statistical test). **(C, D)** Percentages of CD56bright NK cells and CD56dim NK cells in the CLTs and DLTs of hepatic AE patients (n = 10, Wilcoxon non-parametric statistical test). (P values are presented as **P < 0.01), no significant different (ns). CLT, close lesion tissue; AE, alveolar echinococcosis; DLT, distal lesion tissue.

Human NK cells are divided into two subsets, and their main functions are not exactly the same: CD56bright NK cells are responsible for cytokine secretion, whereas CD56dim NK cells are responsible for cytotoxicity (28). We analysed the percentage of NKG2A expression in different subsets of NK cells in hepatic AE patients. The gating strategy used to separate the CD56bright and CD56dim NK cells is shown in **Figure 5A**. We observed that the percentages of total NK cells (**Figure 5B**) and CD56bright NK cells (**Figure 5C**) in the CLT and DLT tissues were not significantly different. However, the percentage of CD56dim NK cells among total NK cells decreased significantly in the CLT (**Figure 5D**). These results further confirmed that the decreased percentage of the NK cell subset was mainly CD56dim NK cells in the CLT tissues of hepatic AE patients.

## Discussion

Our study demonstrated the obviously increased expression of NKG2A in NK cells in the CLT, which may induce a significant reduction in IFN-γ production from closely related lesion tissue; moreover, increased NKG2A expression is correlated with lesion activity and fibrosis in hepatic AE patients. According to present research reports, this is the first report on hepatic NK cells and their related functions in hepatic AE patients.

The percentage of human liver NK cells among total lymphocytes is approximately 25%–40% (29), and approximately 90% of NK cells in the peripheral blood and spleen belong to the CD56dim subset, whereas only 50% of NK cells in the liver belong to the CD56dim subset. NK cell subsets have different effects on the proliferation response, cytotoxicity, cytokine production, and expression of NK cell receptors and adhesion molecules (29). NK cells, as critical components of the innate immune system, are important effector lymphocyte populations involved in antitumour and anti-infection immunity (30). However, in the context of tumours and chronic infections, NK cells exhibit an exhausted status similar to that of exhausted T cells, resulting in poor effector function and an altered phenotype. In ovarian cancer, the expression of NKG2A is upregulated, which induces NK cell failure (28). The upregulation of NKG2A on NK cells may indicate antitumour immune tolerance and promote tumour metastasis in lung cancer patients (31). Previous studies have shown that the NK toxicity of peripheral blood mononuclear cells (PBMCs) in AE patients is lower than that in non-parasitic biliary disease patients (32). Another recent study revealed that the vesicular fluid of *E. multilocularis* has an inhibitory effect on the activation and proliferation of NK cells in human PBMCs (10). According to previous relevant studies and our research, the increased expression of NKG2A on NK cells in the CLT of hepatic AE patients may be the main mechanism mediating NK cell dysfunction. Monalizumab treatment can restore CD107 and IFN-γ production in NK cells against various tumour cells (33). When class I MHC ligands of NK cell inhibitory receptors are downregulated, which commonly occurs in tumour cells, the loss of inhibitory signals and the resulting unabated positive signalling also leads to NK cell activation (IFN-γ and TNF-α). This phenomenon is referred to as the “missing-self” response (34). Therefore, we also demonstrated that the ability of NK cells to secrete IFN-γ and granzyme B in the CLT of a lesion is significantly lower than that in the DLT. Moreover, the percentage of NKG2A+ NK cells in the CLT may be negatively correlated with the percentage of IFN-γ secretion. In other words, the inhibitory molecule NKG2A is upregulated on NK cells in the CLT of the lesion, which is the area with the strongest inflammatory immune response, possibly leading to the downregulation of NK cell function.

Humans are occasional intermediate hosts, and the severity of liver AE in humans is caused by the continuous asexual reproduction of *E. multilocularis* and strong inflammatory granuloma infiltration around it, which leads to pathological damage to the liver after parasitic infection. In this study, we analysed the positive expression of NKG2A in the CLT and DLT surrounding liver AE lesions using immunohistochemical staining techniques. The results suggest that the positive expression of NKG2A in the CLT was significantly greater than that in the DLT and that there may be a positive correlation between NKG2A (CLT/DLT) and PET–CT (SUVmax) values. Reuter (35) used positron emission tomography (PET) to track the metabolic changes in ^18^F-FDG in liver AE lesions, and the active area around the liver AE lesion exhibited differences in energy metabolism. Therefore, at present, PET–CT (SUVmax) values can be used to effectively determine the activity of AE lesions. On the basis of our research results, the greater the expression of NKG2A in the surrounding tissues of the lesion, the greater the activity of the lesion, the significantly lower the degree of fibrosis around the lesion, the weaker the fibrous layer to restrict the lesion, and the greater the speed at which the lesion grows and invades the surrounding normal liver tissue.

In summary, infection of the host with *E. multilocularis* leads to the infiltration of inflammatory cells, proliferation of fibroblasts, and promotion of organ fibrosis. Moreover, the fibrous layer is involved in the inflammatory response around the larvae. On the one hand, this can effectively prevent the parasite from receiving a sufficient immune response from the host, which is beneficial for parasite growth and continued parasitism. On the other hand, it limits the parasite’s continued growth, invasion, and growth of new metastatic lesions. Our research revealed that after infection with *E. multilocularis*, the increased expression of NKG2A on NK cells leads to the downregulation of IFN-γ secretion. The increase in NKG2A expression in the tissue near the lesion in patients with liver AE is similar to that observed in animal experiments, leading to a decrease in the function of NK cells, which secrete IFN-γ and granzyme B, and a reduction in fibrosis around the lesion may increase lesion activity and growth. However, further research is needed to explore the detailed interaction mechanism involved, and we need to expand the sample size to further validate the conclusion.

## Data Availability

The raw data supporting the conclusions of this article will be made available by the authors, without undue reservation.
