# Correction: Hepatic alveolar echinococcosis infection induces a decrease in NK cell function through high expression of NKG2A in patients

**DOI:** 10.3389/fimmu.2025.1638960

**Published:** 2025-10-24

**Authors:** Abuduaini Abulizi, Talaiti Tuergan, Paizula Shalayiadang, Chuanshan Zhang, Ruiqing Zhang, Tiemin Jiang, Qiang Guo, Hui Wang, Liang Li, Renyong Lin, Yingmei Shao, Tuerganaili Aji

**Affiliations:** ^1^ Hepatobiliary & Hydatid Disease Department, Digestive & Vascular Surgery Center, First Affiliated Hospital of Xinjiang Medical University, State Key Laboratory of Pathogenesis, Prevention and Treatment of High Incidence Diseases in Central Asia, Urumqi, China; ^2^ World Health Organization (WHO) Collaborating Center on Prevention and Management of Echinococcosis, Clinical Medicine Institute, First Affiliated Hospital of Xinjiang Medical University, Urumqi, China; ^3^ State Key Laboratory on Pathogenesis Prevention & Treatment of High Incidence Diseases in Central Asia, Xinjiang Medical University, Urumqi, China

**Keywords:** hepatic alveolar echinococcosis, NK cell, NKG2A, immune exhaustion, IFN-γ

Author “Talaiti Tuergan” was erroneously assigned as equal contributing author. The original version of this article has been updated.

